# Integrated Spatial and Single-Cell Transcriptomics Reveals Poor Prognostic Ligand–Receptor Pairs in Glioblastoma

**DOI:** 10.3390/cells14191540

**Published:** 2025-10-01

**Authors:** Makoto Yoshimoto, Kengo Sugihara, Kazuya Tokumura, Shohei Tsuji, Eiichi Hinoi

**Affiliations:** 1 Department of Bioactive Molecules, Pharmacology, Gifu Pharmaceutical University, Gifu 501-1196, Japan; 195130@gifu-pu.ac.jp (M.Y.); 205050@gifu-pu.ac.jp (K.S.); 203016@gifu-pu.ac.jp (K.T.); tsuji-sho@gifu-pu.ac.jp (S.T.); 2 United Graduate School of Drug Discovery and Medical Information Sciences, Gifu University, Gifu 501-1193, Japan; 3 Center for One Medicine Innovative Translational Research (COMIT), Division of Innovative Modality Development, Gifu University, Gifu 501-1193, Japan

**Keywords:** spatial transcriptomics, glioblastoma, cell–cell interaction, gene expression omnibus, the cancer gnome atlas, Chinese glioma genome atlas

## Abstract

**Highlights:**

**What are the main findings?**

- Poor prognostic ligand–receptor pairs are localized in specific regions within glioblastoma tissues.

**What is the implication of the main finding?**

- Our findings provide new insights into pathological cell–cell interactions and treatment for glioblastoma.

**Abstract:**

Glioblastoma (GBM) is an aggressive and lethal malignant brain tumor. Cell–cell interactions (CCIs) in the tumor microenvironment, mediated by ligand–receptor (LR) pairs, are known to contribute to its poor prognosis. However, the prognostic influence of CCIs on patients with GBM and the spatial expression profiles of such LR pairs within tumor tissues remain incompletely understood. This study aimed to identify prognostic LR pairs in GBM and their intratumoral localization via multitranscriptomic analysis. The CCIs among GBM cells as well as between GBM and niche cells were comprehensively evaluated using 40,958 cells in single-cell RNA sequencing datasets. They were found to form intercellular networks in GBM by specific LR pairs, which were mainly implicated in extracellular matrix (ECM)-related biological processes. Survival analysis revealed that 13 LR pairs related to ECM biological processes contributed to poor prognosis (*p* < 0.05, and 95% confidence intervals > 1). Notably, our spatial transcriptomic analysis using three independent GBM cohorts revealed that the identified poor prognostic LR pairs were localized in specific regions within GBM tissues. Although the clinical importance of these LR pairs requires further investigation, our findings suggest potential therapeutic targets for GBM.

## 1. Introduction

Glioblastoma (GBM) is an aggressive and deadly brain malignancy, with a median survival of 15–17 months [1,2]. Although multimodal treatment approaches have been developed, including surgery, radiation therapy, and temozolomide (TMZ) chemotherapy, its treatment outcomes have not improved over the years [3]. The tumor microenvironment (TME) in GBM has complex cellular and non-cellular components. In particular, the TME includes niche cells, such as tumor-associated macrophages/microglia (TAMs), endothelial cells (ECs), cancer-associated fibroblasts (CAFs), and oligodendrocytes (Oligos), as cellular components. Moreover, the TME comprises various non-cellular components, including cytokines, chemokines, growth factors and extracellular matrix (ECM). These various components contribute to tumor progression and therapeutic resistance by interacting with GBM cells and remodeling the TME [4,5,6,7,8,9,10,11]. Thus, a detailed analysis of the cell–cell interactions (CCIs) is required.

Recent advances in single-cell RNA sequencing (scRNA-seq) analysis led to the development of some pipelines that can infer CCIs in TME [12,13,14,15]. Although numerous ligand–receptor (LR) pairs related to poor prognosis among malignant cells and niche cells have been identified in several cancer types [16,17], comprehensive research investigating CCIs related to GBM progress has not yet progressed far. Intracellular signaling events are mediated by secreted factors or molecules expressed on cell membranes that bind to their receptors [18]. The former mechanism mediated by secret factors enables intracellular signaling between spatially distinct cells. On the other hand, the latter requires localization of ligands and receptors in spatial proximity. However, conventional bulk and single-cell analyses involve tissue sample digestion, resulting in the loss of their spatial information on ligand–receptor colocalization within TME.

Spatial transcriptomic (ST) analysis is a revolutionary technology that can overcome the aforementioned challenges by enabling gene expression analysis while preserving tissue morphology [19,20]. ST analysis has already been applied to several cancer types, including GBM, pancreatic cancer, and breast cancer, and its value for intratumoral heterogeneity assessment is increasing [21,22,23]. Therefore, integrating ST analysis with single-cell and bulk transcriptomic analyses may reveal more robust and reliable CCIs. This study aimed to comprehensively investigate the CCIs in GBM cells as well as the interactions between GBM cells and niche cells to identify the LR pairs associated with GBM prognosis and their spatial localization profiles.

## 2. Materials and Methods

### 2.1. scRNA-seq Data Analysis

We used the publicly available gene expression data from patients with GBM (GSE182109) [24] obtained from the National Center for Biotechnology Information’s Gene Expression Omnibus. We selected three newly diagnosed samples (ndGBM-02, ndGBM-04, ndGBM-05) in which all cell types identified in the previous paper were included. The “Seurat” package (ver. 5.3.0) in R software (ver. 4.4.1) was used to analyze downloaded raw datasets. The gene expression data were imported into R software using Read10X function. During pre-processing, cells with less than 500 expressed genes and more than 20% mitochondrial RNA counts were considered to be low-quality cells and then removed using the subset function. Gene expression levels of the remaining cells were normalized using the SCTransform function. To remove batch effects across samples, they were integrated using IntegrationLayers function. For dimensionality reduction, principal component analysis (PCA) and Uniform Manifold Approximation and Projection (UMAP) were performed using the RunPCA and RunUMAP functions. We then computed neighborhood and clustered the data using the FindNeighbors function and FindClusters function. Cell annotation was conducted manually using known marker genes. Copy number variation (CNV) analysis was conducted using “inferCNV” package (ver. 1.22.0) with TAMs regarded as reference for normal cells.

### 2.2. Cell–Cell Interaction Analysis

The “CellChat” package (ver. 2.1.2) was employed to estimate CCIs based on the expression levels of ligands and receptors. The communication strength, which is called communication probability, was calculated with the Hill function. The intensity of the interactions was estimated in the context where GBM cells serve as both signal senders and receivers. Pathway enrichment analysis was conducted with “clusterProfiler” package (ver. 4.14.6) to identify enriched biological processes. The enrichment levels of LR pairs in Reactome pathway were estimated with the compareCluster function.

### 2.3. Bulk RNA-seq Data Analysis

GBM cohorts were downloaded from The Cancer Genome Atlas (TCGA) and Chinese Glioma Genome Atlas (CGGA) databases, respectively. The CGGA and TCGA dataset contained 249 and 391 patients with GBM, and 27 TCGA samples without follow-up data were excluded from survival analysis. Single-sample Gene Set Enrichment Analysis (ssGSEA) was conducted on the TCGA dataset using the “GSVA” package (ver. 2.0.7) with the gsva function. As the ssGSEA method can calculate enrichment scores of gene sets in each sample, it is widely used for sample categorization [25,26,27]. “REACTOME_EXTRACELLULAR_MATRIX_ORGANIZATION” registered in the C2 collection of the Molecular Signatures Database (MSigDB, https://www.gsea-msigdb.org/gsea/msigdb/) was used for calculating ssGSEA score in each GBM sample (accessed on 13 June 2025). Based on the median of ssGSEA score, GBM samples were classified into ECM-High (*n* = 182) and ECM-Low (*n* = 182) groups and conducted gene expression analysis using the “DESeq2” package (ver. 1.46.0) to obtain gene lists for further analysis. Gene Set Enrichment Analysis (GSEA) was conducted between ECM-High and ECM-Low groups using the gene sets from Hallmark and C2 collection of MSigDB with the “clusterProfiler” package (ver. 4.14.6). Gene sets with normalized enrichment score (NES) > 0 and *p* < 0.05 were considered significantly enriched in ECM-High group. The “msigdbr” package (ver. 24.1.0) was used to obtain gene sets for ssGSEA and GSEA.

### 2.4. Survival Analysis of the Patients with GBM and Selection of LR Pairs

The GBM cohorts from TCGA and CGGA databases were used for survival analysis. The patients with GBM were divided into two groups according to the median sum of the identified ligands and receptors expression levels, and then survival analysis was conducted using log-rank test and cox proportional hazards model with “survival” package (ver. 3.6.4). Multiple testing correction was conducted with the Storey method in the “qvalue” package (ver. 2.38.0). We selected LR pairs associated with poor prognosis (*p* < 0.05, hazard ratios (HR) > 1, and 95% confidence intervals (CI) > 1) and significant upregulation compared to normal brain samples.

### 2.5. Spatial Transcriptomics Data Analysis

Three ST data (GSE253080, GSE235672 and GSE194329) [22,28,29] were imported into R software using Read10X function and Read10X_Image function in the “Seurat” package. In pre-processing the dataset, spots with less than 1000 expressed genes and more than 30% mitochondrial transcripts were considered low-quality spots, and then they were removed with the subset function. Gene expression levels of the remaining spots were normalized using the SCTransform function. The following dimensionality reduction and clustering process were conducted with the same pre-processing method of scRNA-seq data. Cluster annotation was conducted manually based on previous histopathological annotation.

### 2.6. Multimodal Intersection Analysis

Multimodal Intersection Analysis (MIA) [30] was conducted, which integrates ST data with scRNA-seq data. This method enables to estimate the enrichment level of each cell type in scRNA-seq data in each cluster within ST data. First, differentially expressed genes (DEGs) in each cell type within the scRNA-seq data (GSE182109) and in each tissue region within the ST data set (GSE253080) were identified using the FindAllMarkers function. Next, the hypergeometric test was conducted to evaluate the significance of overlap between ST and scRNA-seq DEGs with all genes in the ST data set as the gene background to compute *p*-value. On the other hand, we assessed cell type depletion by calculating −log10(1 − *p*). Finally, the calculated enrichment and depletion scores were visualized as a heat map using the “ggplot2” package (ver. 3.5.2).

### 2.7. Ligand-Receptor Colocalization Analysis in GBM Tissue

The expression levels of ligands and receptors for each spatial spot were extracted with the FetchData function. In the case of multiple receptors, their expression levels were summed. For the threshold of expression values, spatial spots with expression levels at or above 80th percentile of all spots per gene were categorized as “Ligand-High” and “Receptor-High”, respectively. The spots satisfying both criteria were categorized as “High-interaction”. These annotations were applied to the GBM tissue images with the SpatialPlot function.

### 2.8. Statistical Analysis

Permutation tests were applied for GSEA and Wilcoxon’s rank-sum test was used to identify DEGs for ST data and scRNA-seq data. For multiple testing correction, we performed Bonferroni’s correction for DEGs analysis and Storey’s correction method for survival analysis. Statistical significance was defined as *p*-value < 0.05.

## 3. Results

### 3.1. Identification of Intercellular Networks in GBM with Bioinformatics Analysis

**Figure 1:**
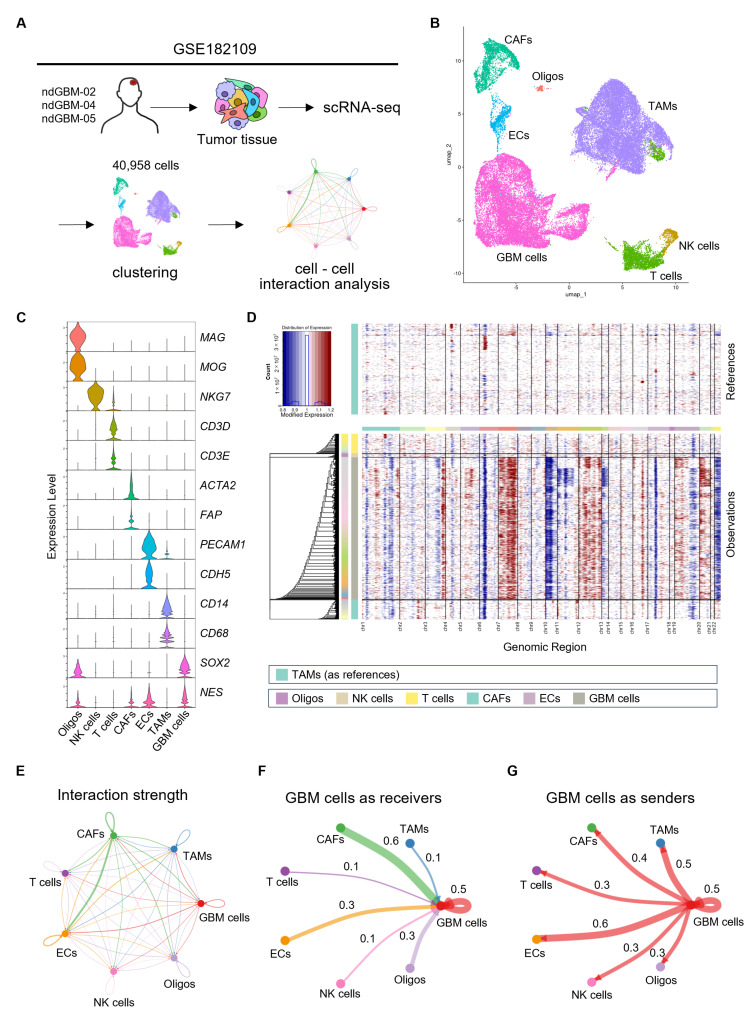
Single-cell analysis reveals intercellular interactions through ligand–receptor signaling. (**A**) Schematic diagram of scRNA-seq data information and cell–cell interactions (CCIs) in GSE182109. (**B**) Uniform manifold approximation and projection (UMAP) showing seven distinct cell populations in GBM tissue. (**C**) Violin plot showing the expression levels of canonical marker genes in each cell type. (**D**) Copy number variation (CNV) profile of different cell types in GBM. Rows correspond to cell types and columns correspond to genomic regions. Red and blue represent copy number gains and losses, respectively. (**E**–**G**) Circle plots indicating CCIs strength among cell types with CellChat pipeline. The line thickness represents CCIs strength calculated based on the ligand and receptor expression levels. Three circle plots indicate: (**E**) CCIs among all cell types, (**F**) CCIs where GBM cells serve as receivers, (**G**) CCIs where GBM cells serve as senders, respectively.

We first analyzed publicly available scRNA-seq datasets from clinical GBM specimens (GSE182109) to profile malignant GBM and other niche cells and to conduct CCIs analysis (Figure 1A). Using a UMAP analysis based on gene expression profiling, we successfully identified seven distinct clusters (Figure 1B). Each cluster was annotated to a specific cell type based on the following canonical marker genes: Oligos [*MAG*, *MOG*], natural killer (NK) cells [*NKG7*], T cells [*CD3D*, *CD3E*], CAFs [*ACTA2*, *FAP*], ECs [*PECAM1*, *CDH5*], TAMs [*CD14*, *CD68*], and GBM cells [*NES*, *SOX2*] (Figure 1C). Furthermore, we conducted CNV inference to validate our classification of GBM cells and non-malignant cells (Figure 1D) and found that GBM cells exhibited the typical CNV pattern in GBM, including chromosome 7 gains and chromosome 10 losses [31]. We also aimed to identify CCIs in GBM using the CellChat pipeline [15], which quantifies the probability of intercellular signaling communication. A Circle plot represented in Figure 1E showed the CCIs among all seven cell types. When GBM cells served as signal receivers, they exhibited the strongest interactions with CAFs (interaction score: 0.6), and then with GBM cells themselves (0.5), ECs and Oligos (0.3), and finally TAMs, T cells, and NK cells (0.1) (Figure 1F). Conversely, in the context where GBM cells served as signal senders, they showed the strongest interactions with ECs (0.6), and then with GBM cells themselves and TAMs (0.5), CAFs (0.4), and finally T cells, NK cells, and Oligos (0.3) (Figure 1G).

These results suggest that GBM cells and niche cells establish intercellular networks in GBM, which may contribute to tumor progression.

### 3.2. Pathway Enrichment Analysis of LR Pairs Activated in GBM

**Figure 2:**
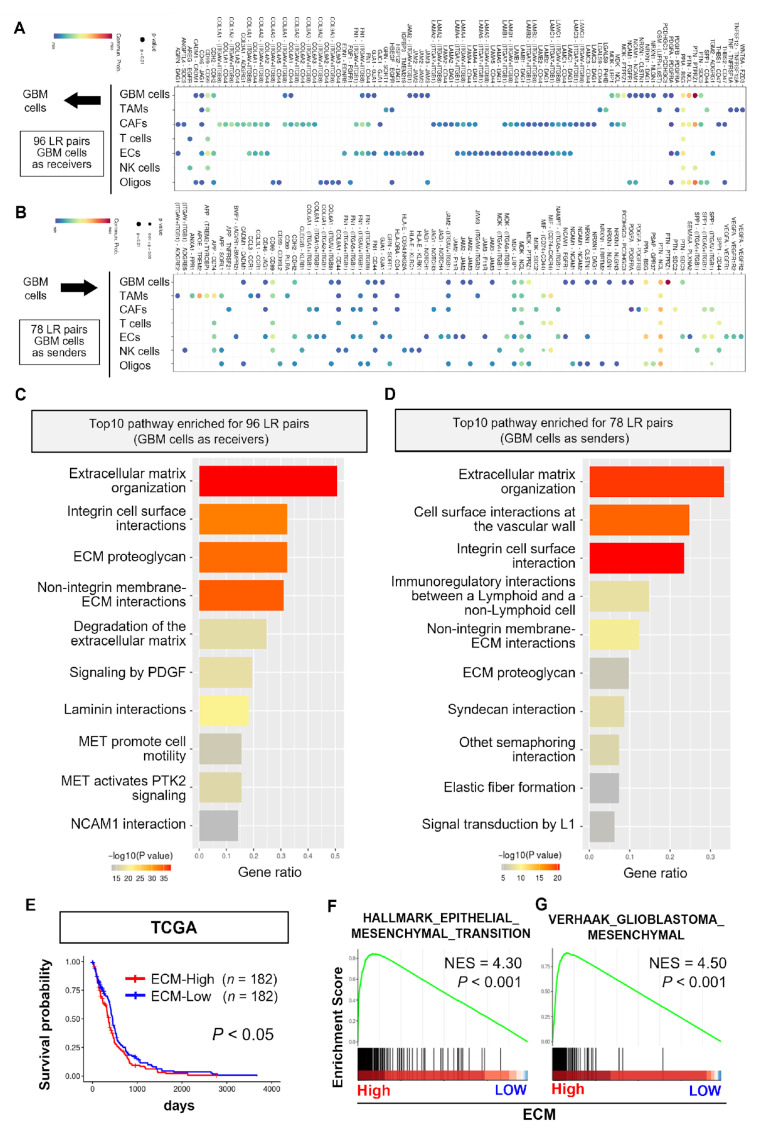
Extracellular matrix-related intercellular interactions are associated with GBM pathogenesis. (**A**,**B**) Bubble plots showing significant ligand–receptor (LR) pairs in the context where GBM cells serve as receivers (**A**) and senders (**B**), respectively. Bubble size indicates *p*-value and color indicates the probability of CCIs. (**C**,**D**) Bar plots indicating the top 10 significantly enriched Reactome pathway of the identified 96 LR pairs (**C**) and 78 LR pairs (**D**), respectively. The bar color represents *p*-value, and the size represents gene enrichment ratios. (**E**) Kaplan–Meier survival analysis for high ECM_signature (*n* = 182) and low (*n* = 182) GBM samples in The Cancer Genome Atlas dataset. (**F**,**G**) Gene Set Enrichment Analysis of epithelial–mesenchymal transition (HALLMARK_EPITHELIAL_MESENCHYMAL_TRANSITION) (**F**) and mesenchymal subtype (VERHAAK_GLIOBLASTOMA_MESENCHYMAL) (**G**), respectively.

GBM cells exhibit autonomous and non-autonomous regulation of tumorigenic potential through CCIs. Using the CellChat pipeline, we identified the specific LR pairs mediating interactions between GBM cells themselves and with niche cells (Figure 2A,B). We identified 96 LR pairs when GBM cells served as signal receivers (Figure 2A) and 78 LR pairs when GBM cells served as signal senders (Figure 2B). Furthermore, we conducted Reactome enrichment pathway analysis to explore the biological functions of the identified LR pairs and demonstrated their enrichment in ECM-related biological processes (e.g., “Extracellular matrix organization”) when GBM cells served both as signal receivers and senders (Figure 2C,D). Previous studies have reported that ECM-related biological processes are closely associated with GBM pathophysiology [32,33]. Kaplan–Meier survival analysis revealed that patients with high ECM_signature had poorer survival (*p* < 0.05) (Figure 2E). Moreover, GSEA revealed that gene sets involved in epithelial–mesenchymal transition and their mesenchymal subtype of GBM cells, which are strongly association with GBM pathogenesis from the perspective of ECM remodeling [34,35], were most enriched in patients with high ECM_signature in Hallmark (NES = 4.30, *p* < 0.001) and C2 collections (NES = 4.50, *p* < 0.001), respectively (Figure 2F,G).

These results indicate that the CCIs in GBM cells and the interaction between GBM cells and niche cells are associated with ECM-related biological processes and may contribute to GBM pathogenesis.

### 3.3. Identification of ECM-Related LR Pairs Associated with Poor Prognosis

**Figure 3:**
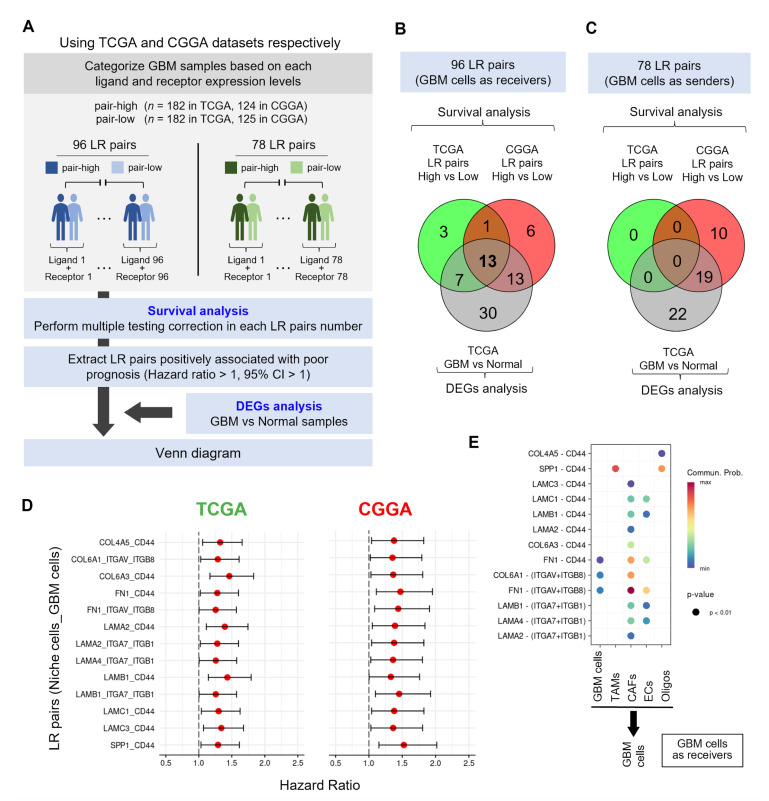
Extracellular matrix-related ligand–receptor interactions predict poor prognosis in patients with GBM. (**A**) Schematic diagram illustrating the identification process of poor prognostic LR pairs. (**B**,**C**) Venn diagram indicating the overlapping LR pairs in the TCGA GBM samples (LR pair-high; *n* = 182 vs. pair-low; *n* = 182 and normal brain samples; *n* = 5 vs. GBM samples; *n* = 386) and CGGA (LR pair-high; *n* = 124 vs. pair-low; *n* = 125). The patients with sum of ligand and receptor expression levels above the median were categorized into LR pair-high groups and the others were categorized into pair-low groups, respectively. (**D**) Forest plot showing hazard ratios for 13 ECM-related LR pairs associated with poor prognostic in the TCGA and CGGA datasets. (**E**) Bubble plot showing the identified 13 LR pairs in the context where GBM cells serve as receivers. The dot size indicates *p*-value and the color indicates the probability of CCIs.

To further clarify the contribution of the identified LR pairs (Figure 2) to GBM progression, we conducted survival analysis using TCGA and CGGA datasets. In each dataset, we evaluated the identified 96 and 78 LR pairs, respectively. For each GBM sample, we summed the expression levels of the ligand and its corresponding receptor for each LR pair and categorized the samples into LR pair-high and pair-low groups based on its median value. After conducting survival analysis between these groups individually for each LR pair, we extracted those that demonstrated positive association with poor prognosis in the LR pair-high groups (*p* < 0.05, HR > 1, 95% CI > 1). In addition, we conducted DEGs analysis on the genes from 96 and 78 LR identified pairs between GBM and normal samples. Then, we selected LR pairs upregulated in GBM samples and then conducted a Venn diagram along with those positively associated with poor prognosis (Figure 3A). Among the 96 LR pairs in the context where GBM cells served as signal receivers, the Venn diagram showed that 13 ECM-related LR pairs were fulfilled with both poor prognosis in the TCGA and CGGA datasets and upregulation in GBM samples (Figure 3B). However, when GBM cells acted as signal senders, no LR pairs associated with poor prognosis were identified in the TCGA dataset; thus, no overlapping LR pairs were detected (Figure 3C). Next, we visualized the 13 LR pairs identified in Figure 3B using forest plots with the TCGA and CGGA datasets (Figure 3D, Table 1). The forest plots showed the identified LR pairs associated with poor prognosis (*p* < 0.05, HR > 1, 95% CI > 1): *Collagen type 4 alpha 5* (*COL4A5*) and *CD44*, *COL6A1* and *Integrin alpha V* (*ITGAV*)/*ITGB8*, *COL6A3* and *CD44*, *Fibronectin 1* (*FN1*) and *CD44*, FN1 and *ITGAV*/*ITGB8*, *Laminin A2* (*LAMA2*) and *CD44*, *LAMA2* and *ITGA7*/*ITGB1*, *LAMA4* and *ITGA7*/*ITGB1*, *LAMB1* and *CD44*, *LAMB1* and *ITGA7*/*ITGB1*, *LAMC1* and *CD44*, *LAMC3* and *CD44*, and *Phosphoprotein 1* (*SPP1*) and *CD44*. The bubble plot showed that GBM cells expressed several ECM receptors, including *CD44* and various integrins, whereas niche cells expressed ECM ligands such as *SPP1*, *LAMA2*, *LAMA4*, *LAMB1*, *LAMC1*, *LAMC3*, *COL4A5*, *COL6A1*, and *FN1* (Figure 3E). Although these ECM ligands are rarely expressed in normal brain tissues, they become upregulated in GBM as a result of ECM remodeling [11].

These results suggest that CCIs through ECM ligands expressed by niche cells and their counterpart receptors expressed by GBM cells are associated with a poor prognosis in GBM.

### 3.4. Spatial Localization of ECM-Related LR Pairs with Poor Prognosis Within GBM Tissues

**Figure 4:**
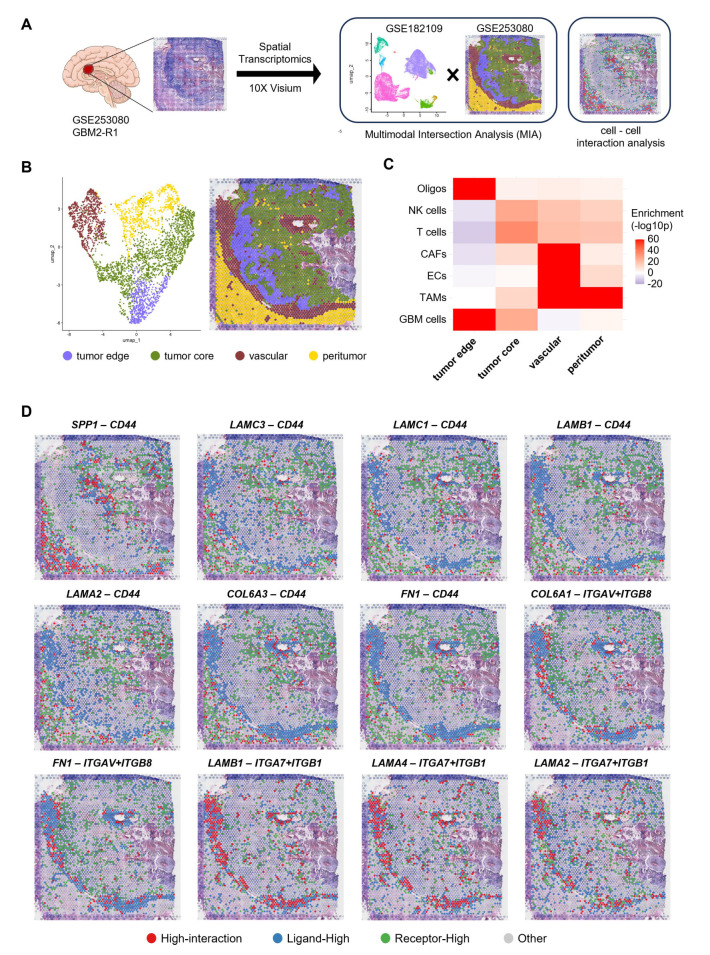
Spatial transcriptomics analysis reveals the localization of extracellular matrix-related ligand–receptor pairs. (**A**) Experimental schemes of spatial transcriptomic analysis using GSE253080. (**B**) UMAP plot (**left**) and spatial localization of the identified clusters (**right**) in the GBM tissue. (**C**) Heatmap of multimodal intersection analysis (MIA) showing correlation significance for the cell types identified in scRNA-seq data (GSE182109) and the clusters (GSE253080). (**D**) Spatial plots showing the localization of the identified ligands and receptors within the GBM tissue. Spatial plots were categorized into four groups based on ligand and receptor expression levels. Spots with their expression levels at or above 80th percentile of all spots per gene were defined as “Ligand-High” and “Receptor-High” spots, respectively. The spots satisfying both criteria were categorized into “High-interaction” spots.

To investigate the spatial localization of the identified LR pairs within GBM tissues, we conducted spatial mapping via ST analysis of a GBM specimen (Figure 4A). Based on a previous report [22], we successfully identified four distinct regions: tumor edge, tumor core, vascular regions, and peritumoral regions (Figure 4B). As one spatial spot contains 2–40 cells, it is inaccurate to annotate specific cell types to certain spatial clusters in ST data. To address this problem, MIA was conducted to estimate the presence probability of specific cell types within certain spatial clusters [30]. A lower *p*-value indicated a higher probability of specific cell types within spatial clusters. The findings that GBM cells were enriched in tumor edge and core whereas ECs were abundant in the vascular region validated our MIA accuracy (Figure 4C). Next, we focused on LR pairs with expression levels at or above the 80th percentile per gene and omitted *COL4A5* from further analysis owing to its low expression level. To reveal the spatial localization of the identified 12 LR pairs within GBM tissues, we categorized spatial spots demonstrating high expression levels of both the ligand and its receptor as high-interaction spots. We identified high-interaction spots with *CD44* as the receptor (*LAMC3-CD44*, *LAMC1-CD44*, *LAMB1-CD44*, *LAMA2-CD44*, *COL6A3-CD44*, *FN1-CD44*, and *SPP1-CD44*) in the tumor core (Figure 4D). Conversely, high-interaction spots with integrins as the receptor (*COL6A1-ITGAV*/*ITGB8, FN1-ITGAV*/*ITGB8*, *LAMB1-ITGA7*/*ITGB1*, *LAMA4-ITGA7*/*ITGB1*, and *LAMA2-ITGA7*/*ITGB1*) were mainly observed in vascular regions and at the interface of vascular regions and tumor edge (Figure 4D).

These findings indicate that ECM-related LR pairs associated with poor prognosis are localized in specific spatial regions within GBM tissues.

### 3.5. Validation of the Spatial Findings Using Independent Visium GBM Cohort

**Figure 5:**
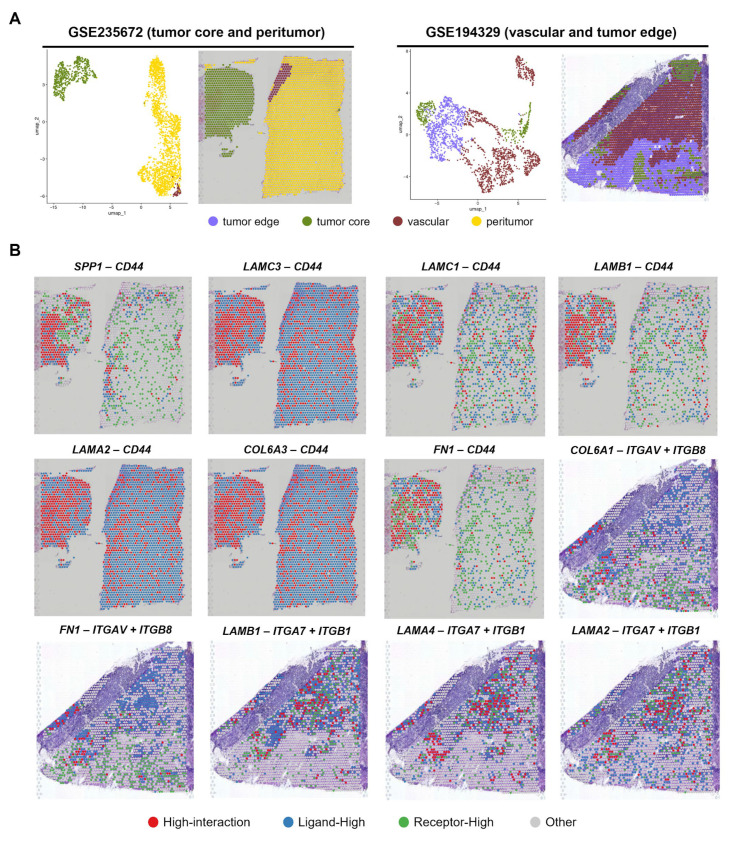
Spatial transcriptomic analyzing of independent GBM cohorts validates the spatial profiles. (**A**) UMAP plot (**left**) and spatial localization of the identified clusters (**right**) in the GBM samples obtained from GSE235672 and GSE194329, respectively. (**B**) Spatial plots demonstrating the localization of the identified LR pairs within the GBM tissues. Spatial plots were classified into four groups based on ligand and receptor expression levels. The categorization criteria are the same as those in Figure 4D.

To confirm the localization of the identified LR pairs shown in Figure 4, we conducted spatial mapping on two additional GBM samples (Figure 5A). As a GBM sample from GSE235672 included tumor core and peritumor regions, we analyzed the expression levels of LR pairs with CD44 as a receptor. Among the seven LR pairs with CD44 as a receptor, some LR pairs (*SPP1-CD44*, *LAMC1-CD44*, *LAMB1-CD44* and *FN1-CD44*) were localized in tumor core, although the other pairs (*LAMC3-CD44*, *LAMA2-CD44* and *COL6A3-CD44*) were not detected owing to the low expression levels of their ligands (Figure 5B). Contrarily, through analysis of a GBM sample from GSE194329 containing vascular, tumor core, and tumor edge, we found that all five LR pairs with integrins as receptors were localized in vascular regions and at the interface between vascular regions and tumor edge (Figure 5B). These results recapitulate the spatial profiles of the LR pairs presented in Figure 4.

These findings suggest that partial LR pairs are validated to be localized in specific regions within GBM tissues.

## 4. Discussion

CD44 is a multifunctional transmembrane protein that plays a role in the proliferation, invasion, metastasis and stemness of malignant cells [36]. It binds various ECM components, such as hyaluronic acid, SPP1, fibronectin, collagen, laminin, and proteoglycan [37]. In GBM cells, ECM-bound CD44 induces γ-secretase–dependent cleavage of its intracellular domain, which activates hypoxia-inducible factor-2α under hypoxia and contributes to GBM progression [38,39]. Since the tumor core is generally hypoxic, the CD44-mediated gene regulatory mechanism may be active in the intratumoral region, where CD44 was found to be upregulated in our study (Figure 4 and Figure 5). Moreover, Previous in vitro analysis suggested that the expression levels of CD44, which is also a representative cancer stem cell marker, increase following TMZ treatment due to the acquisition of cancer stemness properties via Smad/Erk signaling [40].

Integrins are transmembrane receptors consisting of heterodimeric alpha and beta subunits that bind to ECM ligands, such as collagen, fibronectin, and laminin [41]. They transduce intracellular signals through FAK, Shc/MAPK, and Rho-GTPase, which promote cell survival [42]. Although integrin subunits are highly homologous, specific subtypes and their combinations demonstrate distinct or diverse biological activities in GBM cells. Integrin αvβ8 regulates the cellular properties of GBM cells through ECM-dependent Rho-GTPases activities and TGF-β signaling [43,44,45]. Integrin α7β1 enhances the invasiveness and tumorigenesis of GBM cells via FAK signaling [46]. Integrin α5β1 is associated with TMZ resistance through the modulation of the p53 pathway [47]. As ECM deposition in the vasculature of GBM contributes to its stiffness [48,49,50], GBM cells in the tumor edge may exploit these vascular properties through integrins, in a manner distinct from those in the tumor core. Moreover, since certain integrins demonstrate resistance to pharmacological and radiation therapies, they are considered promising therapeutic targets. Cilengitide, an integrin inhibitor targeting the RGD amino acid sequence of integrins, exhibited antitumor efficacy and improvement overall survival [51,52].

Our ST analysis based on the 10X Visium platform has the technical limitation that each spatial spot encompasses multiple cells [53], resulting in lower resolution compared with scRNA-seq. Therefore, we applied MIA to the ST dataset to access cell type enrichment within each annotated region. In recent years, ST analysis at a single-cell resolution has become feasible [54]; thus, in-depth analysis with high-resolution data is useful in gaining deeper insight.

The GBM tissue sample presented in Figure 4 contains blood vessels in the tumor core or peritumor. As hypoxic conditions generally induce angiogenesis [55], the blood vessels present in the tumor core region are likely to have been formed by the tumor. Contrarily, those present in the peritumor region are likely to have originated from brain tissues. GBM cells tap pre-existing ones to obtain oxygen and nutrients for their brain parenchyma infiltration, a phenomenon referred to as vessel co-option [56]. Blood–brain barrier (BBB), which plays a pivotal role in maintaining brain homeostasis, is a major obstacle to GBM treatment. Within the tumor core, BBB disruption may facilitate drug delivery [57]; however, this region is generally necrotic and has low metabolic activity. Contrarily, the function of the BBB is partially or completely preserved in the peritumor region, which results in treatment resistance owing to drug excretion by transporters in addition to its physical barrier function [58].

Although further analysis is warranted to elucidate the LR pairs associated with poor prognosis in GBM, the identified LR pairs may serve as both biomarkers for prognostic stratification and promising molecular targets for therapeutic intervention. Collectively, our findings suggest the potential for novel and effective GBM therapeutic strategies targeting CCIs.

## 5. Conclusions

The findings of the current study suggest that the LR pairs associated with poor prognosis in GBM are localized in specific tumor regions, which provides new insights into potential treatment strategies for GBM.

## Figures and Tables

**Table 1:** The result of hazard ratios and 95% confidence intervals of LR pairs of the two GBM databases.

Ligand	Receptor	HR (TCGA)	95% CI (TCGA)	HR (CGGA)	95% CI (CGGA)
*COL4A5*	*CD44*	1.32	1.05–1.65	1.38	1.03–1.82
*COL6A1*	*ITGAV*/*ITGB8*	1.29	1.02–1.61	1.35	1.02–1.79
*COL6A3*	*CD44*	1.46	1.16–1.82	1.36	1.03–1.80
*FN1*	*CD44*	1.28	1.02–1.60	1.47	1.11–1.95
*FN1*	*ITGAV*/*ITGB8*	1.25	1.00–1.56	1.44	1.08–1.90
*LAMA2*	*CD44*	1.39	1.11–1.74	1.39	1.04–1.84
*LAMA2*	*ITGA7*/*ITGB1*	1.28	1.02–1.60	1.38	1.04–1.82
*LAMA4*	*ITGA7*/*ITGB1*	1.26	1.00–1.57	1.36	1.02–1.80
*LAMB1*	*CD44*	1.43	1.14–1.79	1.33	1.00–1.75
*LAMB1*	*ITGA7*/*ITGB1*	1.26	1.00–1.57	1.45	1.09–1.92
*LAMC1*	*CD44*	1.30	1.03–1.62	1.38	1.04–1.82
*LAMC3*	*CD44*	1.34	1.07–1.67	1.36	1.02–1.80
*SPP1*	*CD44*	1.29	1.03–1.61	1.52	1.14–2.01

## Data Availability

The ST data and scRNA-seq data used in this study are openly available in the Gene Expression Omnibus (https://www.ncbi.nlm.nih.gov/geo/, accessed on 23 April 2025) database. The public GBM bulk RNA-seq data is available in TCGA (https://portal.gdc.cancer.gov/) and CGGA (https://www.cgga.org.cn/) database.

## Abbreviations

The following abbreviations are used in this manuscript:

CAFs	Cancer-associated fibroblasts
CCIs	Cell–cell interactions
CGGA	Chinese glioma genome atlas
CNV	Copy number variation
ECM	Extra-cellular matrix
ECs	Endothelial cells
GBM	Glioblastoma
GSEA	Gene set enrichment analysis
LR	Ligand-receptor
MIA	Multimodal intersection analysis
MsigDB	Molecular signature database
NK cells	Natural killer cells
Oligos	Oligodendrocytes
PCA	Principal component analysis
ST	Spatial transcriptome
TAMs	Tumor-associated macrophages/microglia
TCGA	The cancer genome atlas
TME	Tumor microenvironment
UMAP	Uniform Manifold Approximation and Projection
scRNA-seq	Single cell RNA sequence
ssGSEA	Single sample gene set enrichment analysis
